# Can the Ratio SUVmax of the Lesion/SUVmax of Mediastinal Tissues Guide the Choice of Surgical Access for the Resection of Thymic Epithelial Tumors?

**DOI:** 10.3389/fsurg.2022.852906

**Published:** 2022-03-15

**Authors:** Sotirios D. Moraitis, Apostolos C. Agrafiotis, Evangelia Skoura, Dimitrios Kalkanis, Dimitrios Moraitis, Periklis Tomos, Theodoros Liakakos, Dimitrios Angouras

**Affiliations:** ^1^Department of Joint Corps Armed Forces Cardiac Surgery, 401 Hellenic Army Hospital, Athens, Greece; ^2^Department of Thoracic Surgery, Athens Naval and Veterans Hospital, Athens, Greece; ^3^Department of Thoracic Surgery, Saint-Pierre University Hospital, Université Libre de Bruxelles (ULB), Brussels, Belgium; ^4^Department of Nuclear Medicine, Bioiatriki, Athens, Greece; ^5^Department of Nuclear Medicine, Iatropolis, Athens, Greece; ^6^Department of Administration, Athens Naval and Veterans Hospital, Athens, Greece; ^7^Department of Thoracic Surgery, Attikon University Hospital, Medical School, National and Kapodistrian University of Athens, Athens, Greece; ^8^Department of Surgery, Laikon University Hospital, Medical School, National and Kapodistrian University of Athens, Athens, Greece; ^9^Department of Cardiac Surgery, Attikon University Hospital, Medical School, National and Kapodistrian University of Athens, Athens, Greece

**Keywords:** standard uptake value, surgical access, thymic epithelial tumors, thymoma, thymic carcinoma

## Abstract

**Background:**

There are studies showing the utility of the 18-fluorodeoxyglucose positron emission tomography (^18^FDG PET) scan in the management of patients with thymic epithelial tumors. It seems to be a correlation between the standard uptake value (SUVmax) of thymic epithelial tumors and the histological type and the stage. This study aims to use the ratio of the SUVmax of the lesion to the SUVmax of the adjacent mediastinal tissues in order to guide the choice of the surgical access.

**Methods:**

All patients who presented an anterior mediastinal lesion with a high suspicion of being of thymic origin were included in a prospective database. A ratio inferior to 1 could predict a benign nature and less aggressive behavior, and a minimally invasive approach was performed. A ratio superior to 1 suggested a malignant and aggressive behavior, and a median sternotomy (or a thoracotomy) was performed.

**Results:**

There were 15 male (mean age 44.6 ± 16.26 years, range 25–73) and 15 female patients (mean age 50.1 ± 16.94 years, range 25–76). When the ratio is inferior to 1, it predicts benign disease in 80% of cases. When it is superior to 1, it predicts in half of cases advanced histological types (high risk thymomas and thymic carcinomas). On the contrary, it can quite accurately predict advanced Masaoka–Koga stages.

**Conclusions:**

The protocol of this study is in accordance with the current literature showing the utility of ^18^FDG PET scan in the treatment of thymic epithelial tumors. This study goes one step further since the choice of surgical access is based on the SUVmax values. The ratio SUVmax of the lesion/SUVmax of the mediastinal tissues could be a new marker, more pertinent than absolute SUVmax values.

## Introduction

Thymic epithelial tumors consist a highly heterogeneous group of anterior mediastinal lesions. They account for 15% of all anterior mediastinal tumors (1). Except for this group of tumors, there are also benign lesions of thymic origin, such as thymolipoma, thymic cysts, and thymic hyperplasia, which are treated with surgical resection (1). Thymic cysts can be congenital or acquired, the latter carrying a greater risk to be associated with inflammatory conditions or neoplasms, such as thymoma, thymic carcinoma, and Hodgkin's lymphoma (1). On the other hand, the diagnosis of thymic hyperplasia is important for two reasons. First, it must be distinguished from thymic neoplasia, and secondly because it can be associated with autoimmune conditions, such as myasthenia gravis and rheumatoid arthritis (2). Thymomectomy plus total thymectomy (thymothymectomy) is recommended over simple thymomectomy for the surgical treatment of thymic epithelial tumors (3). The optimal surgical access is a subject of debate (4). However, it would be useful for the surgical planning to be able to predict the nature (especially in terms of invasiveness) of the lesion. There is much research during the last years concerning the utility of the 18-fluorodeoxyglucose positron emission tomography (^18^FDG PET) scan (5–15). It seems to be a correlation between the standard uptake value (SUVmax) of thymic epithelial tumors and the histological type according to the WHO classification and the stage according to the Masaoka–Koga classification. There are also studies trying to correlate the ratio of the SUVmax of the lesion to the SUVmax of the adjacent mediastinal tissues with the invasive nature of thymic epithelial tumors. Based on those data, this study aims to use this ratio in order to guide the choice of the surgical access for the resection of these lesions.

## Methods

This is a prospective single-center study. The local ethics committee approved the study protocol (Ref: 1722/23-05-2014). From June 2014 to June 2020, all patients who presented with an anterior mediastinal lesion with a high suspicion of being of thymic origin were included in a prospective database. Patients who were considered functionally inapt to undergo surgery, patients with lesions that were considered not resectable, and patients who declined surgical treatment were excluded from the study. Informed consent was obtained from all patients. Preoperatively, all patients underwent pulmonary function tests, a cardiac ultrasound, and a chest CT scan with contrast agent injection (if no contra-indication). The presence of antibodies against the acetylcholine receptor (anti-AChR Ab) and anti-muscle-specific kinase antibodies (anti-MuSK Ab) in the plasma of patients was investigated. A chest MRI was not routinely performed. An ^18^FDG PET scan was performed in all patients as part of the standard preoperative workup. The SUVmax of the lesion was calculated by the nuclear medicine physician, and the SUVmax of the mediastinum was by convention calculated at the level of the aortic arch. According to our protocol, a ratio inferior to 1 could predict a benign nature and less aggressive behavior of the lesion; if there was no other contra-indication or technical difficulty (e.g., a voluminous lesion), then a minimally invasive approach, such as thoracoscopic thymectomy or cervical thymectomy, was performed. On the contrary, a ratio superior to 1 could suggest a malignant and aggressive behavior; in that case, a median sternotomy (or a thoracotomy if the tumor was lateralized) was the preferred surgical access. In case of a perioperative modification of the surgical access (e.g., conversion to a full median sternotomy in case of hemorrhage, technical difficulties, or if locally invasive disease was encountered during a minimally invasive approach), the patients were analyzed in an intention-to-treat manner.

## Statistical Analysis

The chi-square test was used for the identification of independence between two nominal variables. The Fisher criterion was applied. In case of association of two variables, the logistic regression technique was used. Logistic regression examines a depended nominal variable regarding one or more independent ones. In particular, a logistic regression model has been implemented to measure the relationship between diameter and SUVmax ratio superior to 1. A Mann–Whitney *U*-test was used as a non-parametric alternative to the independent *t*-test. The chi-square test and the logistic regression technique examined the dependency or independency among variables. IBM SPSS 23 and Microsoft Office suite (Excel) spreadsheets were used to analyze and graph the data.

## Results

**Figure 1:**
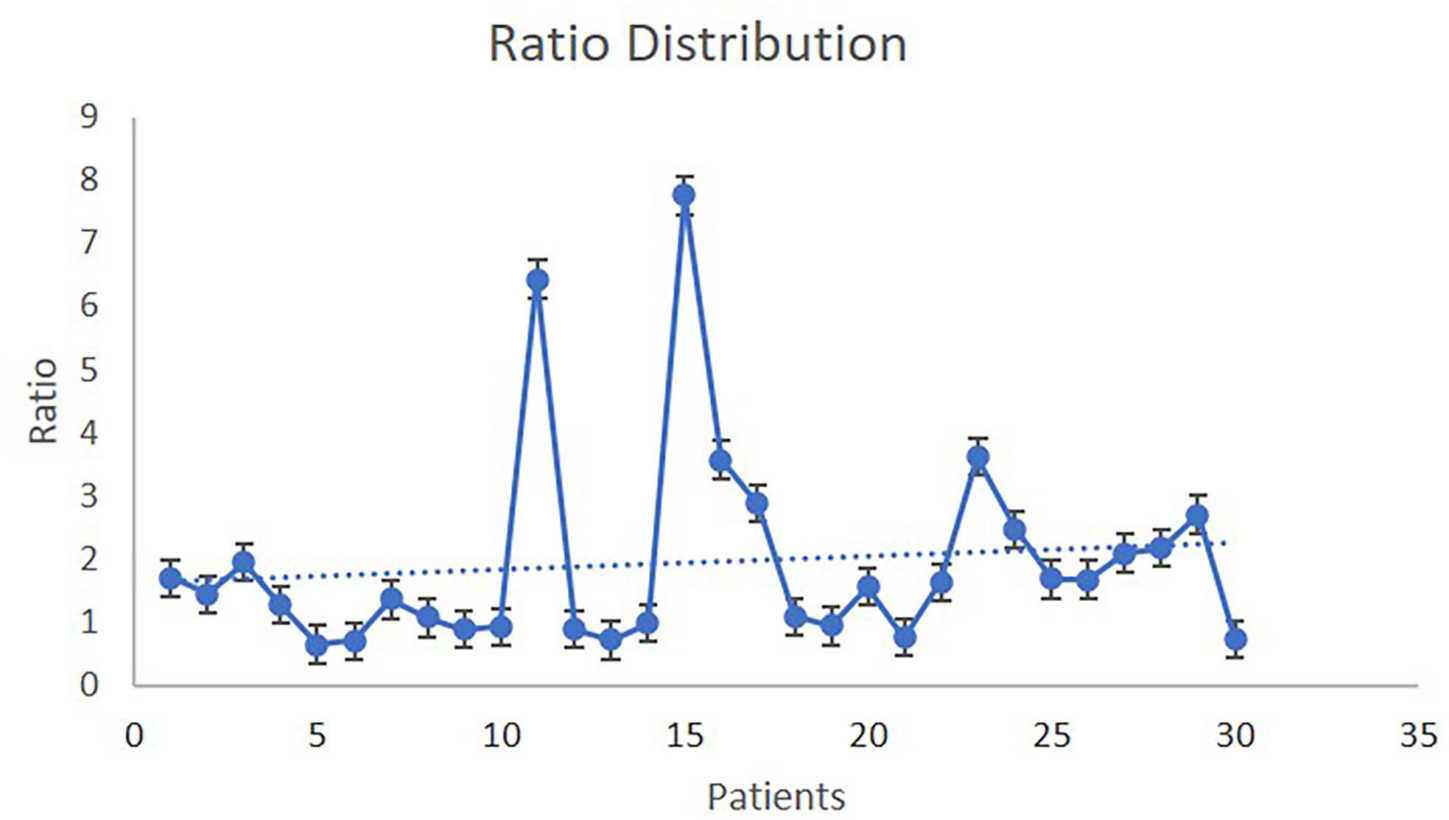
The SUVmax of the lesion/SUVmax of mediastinal tissue ratio distribution.

Thirty patients were included in this protocol (mean age 47.4 ± 16.55 years, range 25–76). There were 15 male (mean age 44.6 ± 16.26 years, range 25–73) and 15 female patients (mean age 50.1 ± 16.94 years, range 25–76). Three patients (10%) suffered from myasthenia gravis. The demographic and other patients' characteristics are demonstrated in Table 1. In 10 patients, the ratio SUVmax of the lesion/SUVmax of the mediastinal tissues was inferior to 1 (Group 1), and in 20 patients, the ratio was superior to 1 (Group 2). The ratio distribution is demonstrated in Figure 1. The characteristics of each group according to the SUVmax ratio are demonstrated in Table 2. In the Group 1, three patients underwent a median sternotomy. In fact, two of the patients declined minimally invasive surgery and preferred a sternotomy, and the third patient presented with a lesion of 10 cm. In the whole group of 10 patients, the histopathological examination of the resected specimen revealed 8 benign lesions and 2 thymomas with the WHO classification histological type superior to B1 and Masaoka–Koga stage superior to I. In the Group 2, one patient underwent VATS (she refused a median sternotomy), and another patient underwent cervical thymectomy (she had already undergone a total thyroidectomy for thyroid cancer, and she refused another surgical access than a collar incision). In the whole group of 20 patients, the final histology was compatible with high-risk thymomas (thymomas with the WHO classification histological type superior to B1) in 8 patients. The Masaoka–Koga stage was I in 2 cases and superior to I in 11 cases. As shown in Table 2, there are more thymomas and more advanced Masaoka–Koga stages in the SUVmax ratio >1 group; nevertheless, the small enrollment does not permit to reach statistical significance. In half of the patients of this cohort, there was a capsular invasion (15 out of 30 patients). More specifically, the 60.9% of patients operated with an open procedure presented a capsular invasion. On the contrary, only 14.3% of patients who underwent minimally invasive surgery had a capsular invasion. In the SUVmax ratio >1 group, the capsular invasion was significantly higher (*p* = 0.02). To estimate the relationship between “SUV ratio” and “presence of malignancy,” the technique of logistic regression was used. The related odds ratio is 12. The conversion of odds ratio to probability reveals that the probability of presence of malignancy with ratio >1 is 92.31%. In addition, the logistic regression model revealed that if the diameter of the tumor is increased per 1 cm, then the odds ratio of presence in the Group 2 (SUVmax ratio > 1) is 1.719 higher.

**Table 1:** Demographics and other patients' characteristics.

Sex (M/F)	15/15
Age (mean ± SD) [range] years	(47.4 ± 16.55) [25–76]
Size (mean ± SD) [range] cm	(7.92 ± 3.41) [2.2–18]
Thymomas	14 (46.6%)
Thymic carcinomas	2 (6.6%)
Thymolipomas	3 (10%)
Thymic hyperplasia	7 (23.3%)
Thymic remnant	3 (10%)
Other	1 (3.3%)
SUVmax of the lesion (mean ± SD) [range]	(3.47 ± 2.39) [1.1–12.4]
SUVmax of mediastinal tissues (mean ± SD) [range]	(1.89 ± 0.41) [1.3–3.3]
SUVmax ratio (mean ± SD) [range]	(1.96 ± 1.62) [0.65–7.75]
**WHO Classification**	
A	1 (7.1%)
AB	3 (21.4%)
B1	2 (14.2%)
B2	3 (21.4%)
Mixed B1B2	1 (7.1%)
B3	–
Mixed B2B3	4 (28.5%)
**Masaoka–Koga Classification**	
I	2 (12.5%)
IIa	5 (31.2%)
IIb	3 (18.7%)
III	3 (18.7%)
IVa	3 (18.7%)
**Surgical access**	
Sternotomy	19 (63.3%)
VATS	8 (26.6%)
Thoracotomy	2 (6.6%)
Cervicotomy	1 (3.3%)

**Resection margins (thymomas and thymic carcinomas)**	
R0	13 (81.3%)
R1	3 (18.7%)

*VATS, video-assisted thoracoscopic surgery*.

**Table 2:** Comparison of the two groups of patients according to the SUVmax ratio.

**Category**	**Group 1 (SUVmax ratio <1)**	**Group 2 (SUVmax ratio >1)**	***p*-value**
Sex (M/F)	(7/3)	(8/12)	0.245
Age (mean ± SD) [range] years	42.6 ± 15.76 [27–73]	49.8 ± 16.8 [25–76]	0.373
Size (mean ± SD) [range] cm	(5.79 ± 2.49) [2.2–10]	(9.09 ± 3.25) [4.5–18]	0.002
Thymomas	2	12	0.058
Thymic carcinomas	0	2	0.54
Thymolipomas	3	0	0.03
Thymic hyperplasia	3	4	0.657
Thymic remnant	2	1	0.251
Other	0	1	1
**WHO classification**		
A	0	1	1
AB	0	3	0.532
B1	0	2	0.54
B2	1	2	1
Mixed B1B2	0	1	1
B3	0	0	–
Mixed B2B3	1	3	1
**Masaoka**– **Koga classification**		
I	0	2	0.54
IIa	1	4	0.64
IIb	1	2	1
III	0	3	0.532
IVa	0	3	0.532
**Surgical access**		
Sternotomy	3	16	0.005
VATS	7	1	0
Thoracotomy	0	2	0.54
Cervicotomy	0	1	1
**Resection margins** **(thymomas and thymic carcinomas)**		
R0	2	11	0.119
R1	0	3	0.532
Capsular invasion	2	13	0.02

*VATS, video-assisted thoracoscopic surgery*.

## Discussion

There are many studies that investigated the utility of ^18^FDG PET scan in the workup of thymic epithelial tumors in terms of diagnosis, evaluation of aggressive behavior, and response to treatment (5–15). Kumar et al. (5) stressed the possibility to differentiate low-risk (A, AB, and B1) and high-risk (B2 and B3) thymomas according to the WHO classification by using the ^18^FDG PET/CT scan. Thymomas can also be distinguished from thymic carcinomas. The studies conducted by Sung et al. (6) and Luzzi et al. (7) were in the same direction. In addition, they confirmed the opinion of El-Bawab et al. (8) concerning the contribution of ^18^FDG PET scan to the differential diagnosis between thymomas and thymic hyperplasia. Igai et al. (9) were able to correlate the SUVmax of the tumor with the histological type, but they did not correlate the SUVmax with the stage of the disease. Similarly, Purandare et al. (14) showed that the SUVmax of thymic carcinomas was significantly higher than low-risk and high-risk thymomas. The SUVmax in patients with advanced stage disease was higher but not statistically significant compared to the early-stage disease (14). On the contrary, Fukumoto et al. (10) and Ito et al. (15) proved this correlation. Furthermore, the opinion of Ito et al. (15) is that there is a statistically significant difference in the SUVmax between thymic carcinoma and high-grade thymoma, among high-grade and low-grade thymomas, and that the ^18^FDG PET/CT scan could predict tumor invasion into pericardium, lungs, and brachiocephalic vein (15). Watanabe et al. (16) presented that the ^18^FDG PET scan can differentiate thymoma from thymic cancer, diffuse large B-cell lymphoma, and Hodgkin's lymphoma, whereas the SUVmax of the lesion cannot predict the histological diagnosis of thymoma (16). Terzi et al. (11) evaluated the SUVmax of the lesion, the SUVmax of the mediastinum, and the ratio of these two variables. They found that there is a correlation between this ratio and the advanced stage of the disease (11). A meta-analysis showed that SUVmax could be able to predict the WHO grade in thymic epithelial tumors (17). On the contrary, there is no robust data permitting to use FDG uptake as a predictive factor for disease stage.

**Figure 2:**
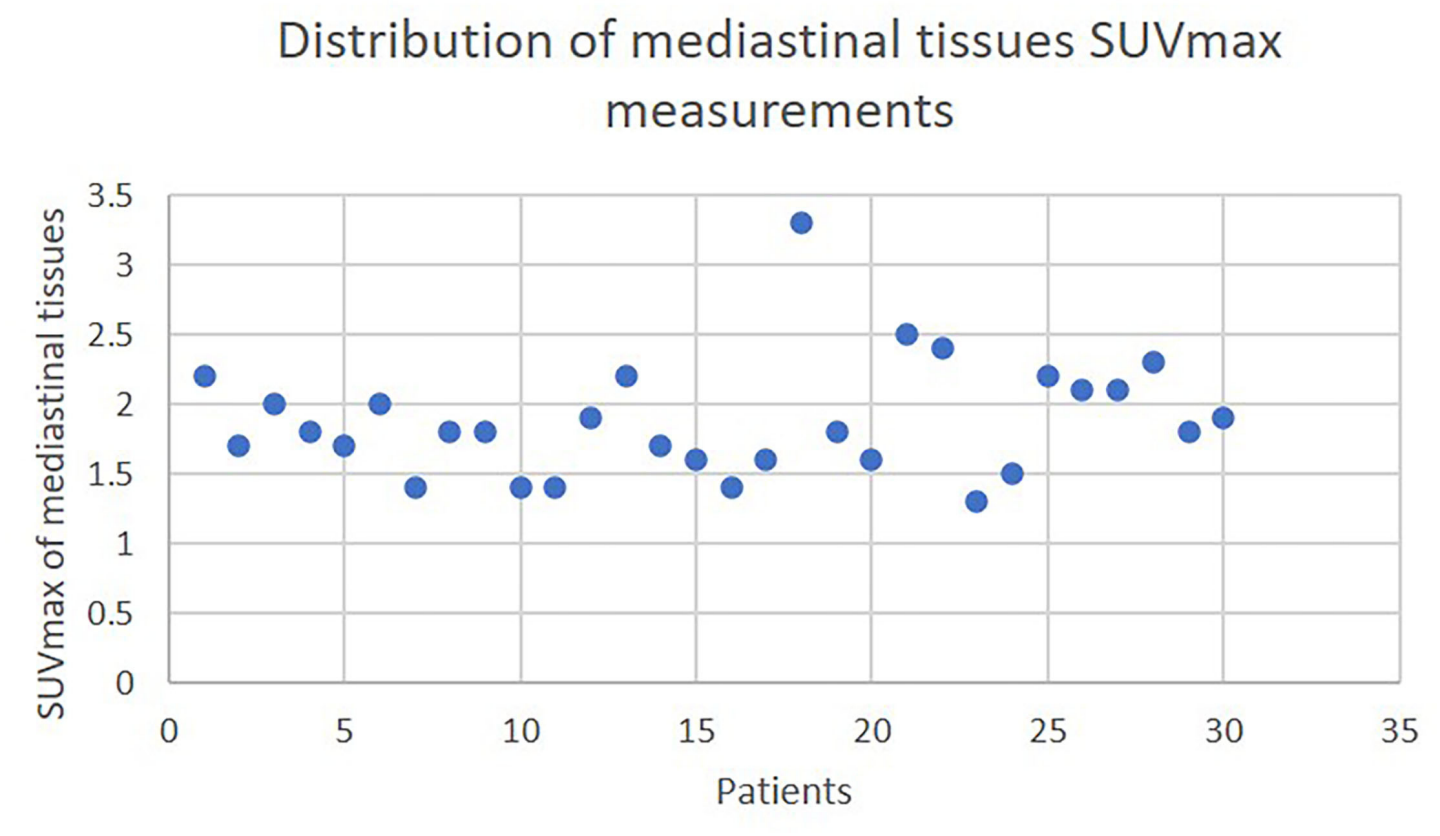
The distribution of mediastinal tissue SUVmax.

To the best of our knowledge, there is no previous study that takes into consideration the values of the ^18^FDG PET scan to guide the choice of the surgical access. The choice of the surgical access is of paramount importance, especially in case of advanced Masaoka–Koga stage, in order to achieve free surgical margins. Since there is no evidence regarding the optimal surgical access for thymic epithelial tumors (18–20), its choice should be based on tumor size, surgical expertise, and patient's preference. In the protocol presented herein, all these elements were taken into account while choosing the surgical access, justifying the derogations from the initial hypothesis. In our study when the ratio SUVmax of the lesion/SUVmax of the mediastinal tissues is inferior to 1, it seems to predict benign disease in 80% of cases. Consequently, a minimally invasive approach is privileged even if it has some limitations, as in case of a voluminous lesion. On the other hand, the choice of an open sternotomy may seem maximalist because there are some false positives in that group. Nevertheless, when the ratio is superior to 1, the probability of presence of malignancy is 92.31%, whereas it can quite accurately predict advanced Masaoka–Koga stages. It can predict in half of cases advanced histological types according to the WHO classification (high-risk thymomas and thymic carcinomas). Taking into account that nowadays all types of thymomas regardless of histological type are considered as malignant lesions (21–24) in contrast to the previous belief that A, AB, and B1 types are rather benign, then a ratio >1 in the current study predicted malignancy in 15 out of 20 cases. The selection of an open access procedure, based on ratio >1, led us on the percentage of 81.3% of R0 resection margins (Table 1). Apart from the above limitations, the most important is the fact that it is a single-center cohort with a small enrollment. Nevertheless, this protocol can be the basis for the conduction of larger multicentric studies in order to validate the usefulness of the ratio. The strong point of this study is its prospective nature. In addition, the use of the ratio rather than absolute SUVmax values could overcome the potential confounding factors related to SUVmax variations due to physiological and technical factors. Van Den Hoff et al. (24) strongly support the opinion of the use of tumor-to-blood pool ratio, instead of tumor SUV, for SUV-based approaches. In our study, the narrow range of the denominator's measurements (range of SUVmax of mediastinal tissues: 1.3–3.3) could allow us a speculation about the pertinence of the ratio, as an index (Figure 2).

## Conclusions

Despite its small enrollment, this study predicts quite accurately the behavior of thymic epithelial tumors. The protocol of this study is in accordance with the current literature showing the utility of ^18^FDG PET scan in the treatment of thymic epithelial tumors. This study goes one step further since the choice of surgical access is based on the SUVmax values. In particular, the ratio SUVmax of the lesion/SUVmax of the mediastinal tissues could be a new marker, more pertinent than absolute SUVmax values. Nevertheless, other parameters, such as tumor size, patient preference, experience of the operating surgeon, and local infrastructure, should be considered while planning the surgical access. Larger studies should be conducted to validate such a protocol in the highly heterogeneous environment of thymic epithelial tumors.

## Data Availability Statement

The raw data supporting the conclusions of this article will be made available by the authors, without undue reservation.

## Ethics Statement

The studies involving human participants were reviewed and approved by Athens Naval and Veterans Hospital Ethics Committee (Ref: 1722/23-05-2014). The patients/participants provided their written informed consent to participate in this study.

## Author Contributions

SM, DA, and TL: conception and design. DM, DK, and ES: administrative support. AA and SM: provision of study materials or patients. SM, PT, DK, ES, and TL: collection and assembly of data. SM, DM, AA, and PT: data analysis and interpretation. All authors: manuscript writing and final approval of manuscript. All authors contributed to the article and approved the submitted version.

## Conflict of Interest

The authors declare that the research was conducted in the absence of any commercial or financial relationships that could be construed as a potential conflict of interest.

## Publisher's Note

All claims expressed in this article are solely those of the authors and do not necessarily represent those of their affiliated organizations, or those of the publisher, the editors and the reviewers. Any product that may be evaluated in this article, or claim that may be made by its manufacturer, is not guaranteed or endorsed by the publisher.
